# Assessment of adjunct cognitive functioning through intake interviews integrated with natural language processing models

**DOI:** 10.3389/fmed.2023.1145314

**Published:** 2023-04-21

**Authors:** Toshiharu Igarashi, Yumi Umeda-Kameyama, Taro Kojima, Masahiro Akishita, Misato Nihei

**Affiliations:** ^1^Department of Human and Engineered Environmental Studies, The University of Tokyo, Kashiwa, Japan; ^2^Department of Geriatric Medicine, The University of Tokyo, Bunkyo-ku, Tokyo, Japan; ^3^Institute of Gerontology, The University of Tokyo, Bunkyo-ku, Tokyo, Japan

**Keywords:** gerontology, intake interview, natural language processing, data augmentation, cognitive function

## Abstract

In this article, we developed an interview framework and natural language processing model for estimating cognitive function, based on an intake interview with psychologists in a hospital setting. The questionnaire consisted of 30 questions in five categories. To evaluate the developed interview items and the accuracy of the natural language processing model, we recruited participants with the approval of the University of Tokyo Hospital and obtained the cooperation of 29 participants (7 men and 22 women) aged 72–91 years. Based on the MMSE results, a multilevel classification model was created to classify the three groups, and a binary classification model to sort the two groups. For each of these models, we tested whether the accuracy would improve when text augmentation was performed. The accuracy in the multi-level classification results for the test data was 0.405 without augmentation and 0.991 with augmentation. The accuracy of the test data in the results of the binary classification without augmentation was 0.488 for the moderate dementia and mild dementia groups, 0.767 for the moderate dementia and MCI groups, and 0.700 for the mild dementia and MCI groups. In contrast, the accuracy of the test data in the augmented binary classification results was 0.972 for moderate dementia and mild dementia groups, 0.996 for moderate dementia and MCI groups, and 0.985 for mild dementia and MCI groups.

## 1. Introduction

Dementia is defined as “a chronic decline or loss of various cognitive functions, resulting in the inability to lead a normal daily or social life,” and is an acquired cognitive disorder (1). Cognitive functions are essential for planning and carrying out daily activities such as cleaning, washing clothes, eating, and going out (2). Therefore, people with dementia are unable to make plans and perform routine activities, which can seriously interfere with their daily lives (3). Approximately 10% of the total population will develop this disorder at some point in their lives, and it is generally considered to be a consequence of aging (4). One person develops dementia every 3 s worldwide, and this number almost doubles every 20 years, with the total number of persons with dementia estimated to reach 152 million by 2050 (5).

However, as medical science is yet to find a cure for dementia, it is imperative to detect the trend of cognitive decline as early as possible and intervene at an early stage, as in the case of cancer (6, 7). Positron emission tomography (PET) and magnetic resonance imaging (MRI) are commonly used to test for dementia, but they are not only time-consuming but also require expensive testing equipment (8). Other methods, such as the Mini-Mental State Examination (MMSE) and Hasegawa Dementia Scale (HDS-R), are quick and easy assessment methods; however, because the questions on the test form are fixed, they can be memorized by the examinee, making the tests unsuitable for periodic monitoring (9, 10). In addition, the knowledge that their cognitive function is being tested places a mental burden on those being tested. Many older people refuse to be tested for dementia, and it has been reported that 16% of people with Alzheimer’s disease show distress-fueled reactions such as anxiety, anger, and refusal during testing (11).

Therefore, assessment for dementia based on the verbal abilities of older people has recently attracted attention. Such assessment is not physically invasive and does not require spending long durations of time in a medical facility. In addition, because cognitive function can be monitored periodically, this technique may make it possible to detect changes in cognitive function over time, attracting attention in the medical and research communities.

## 2. Related studies

In general, patients with dementia have reduced language ability compared to healthy controls. Several studies have screened for dementia based on language ability. Studies focusing on language began with the Nun Study in 1996 (12), and the number of studies using machine learning to discriminate patients with dementia from healthy controls has been gradually increasing. According to previous systematic reviews, machine learning-based assessments of cognitive function can be broadly classified into four categories according to the types of features employed: (i) linguistic, (ii) acoustic, (iii) images/movie, and (iv) other types of features such as expressive features or features that depict specific shapes (13, 14). This study focuses on linguistic features that can deal with fillers and feature words in classification tasks.

### 2.1. Classification of cognitive functions by linguistic features

There are mainly three types of analyses for extracting linguistic features: (1) primary lexical-level analysis, (2) semantic analysis, and (3) sentence-level syntactic analysis.

Automated primary lexical analysis (i.e., lexical or word-level analysis) can produce objective linguistic indices and provide valuable insights into cognitive functions. In its most basic form, the text body is treated as a bag of words. That is, the order of words in the text is not considered.

Jarrold et al. used speech data from healthy participants (*n* = 23) and patients with dementia (*n* = 22) to extract part-of-speech counts, semantic density, and word industry classification [using the Linguistic Inquiry and Word Count (LIWC) tool], which were used as features for machine learning (15). Asgari et al. reported that speech data (daily conversation) obtained from people with mild cognitive impairment (*n* = 14) and normal participants (*n* = 21) could be classified as healthy control and mild cognitive impairment (MCI) with up to 73% accuracy using LIWC (16), and LIWC could discriminate HC and MCI with up to 84% accuracy (17).

Fraser et al. (18) used several key lexical features in their analysis of patients with Alzheimer’s disease (AD), wherein the authors used the type-token ratio (TTR) as measures of lexical diversity and richness to discriminate between healthy older controls (*n* = 16) and a small sample of patients with AD (*n* = 8). In addition, they examined the use of other parts of speech (nouns, pronouns, adjectives, and verbs). In particular, the TTR, BI, oscillation, and adjective rates all showed strong group differences between patients with AD and healthy controls, and group classification was achieved with 87.5% cross-validation accuracy.

For semantic analysis, the semantic similarity of natural languages is usually measured computationally by embedding text into a high-dimensional vector space representing its semantic content. The notion of the distance between vectors can then be used to quantify the semantic similarity or difference between words or sentences represented by the vector embedding.

Snowdon et al. calculated semantic density (the number of propositions in a sentence divided by the number of words) and grammatical complexity from autobiographies written by 93 nuns in their 20s (12). They showed that lower semantic density and grammatical complexity in adolescence were associated with lower cognitive function later in life and reported a certain relationship between these values and cognitive function. Kemper et al. also reported that grammatical complexity decreased with age, regardless of the presence of dementia, but semantic density decreased only in the dementia group (19).

Sentence-level parsing can also provide important insights into the cognitive function of the word order in sentences and sentences in paragraphs. For free speech, we need to determine not only which words best convey ideas but also the order in which words form sentences. The complexity of the sentences we produced provides clues to cognitive linguistic health. This section outlines various methods used to measure syntactic complexity as a proxy for cognitive health. Many common structural measures of language are easy to compute, such as average clause length, average sentence length, and the ratio of the number of clauses to the number of sentences.

Orimayre et al. extracted several syntactic and lexical features from a corpus consisting of patients with dementia (*n* = 314) and healthy participants (*n* = 242) provided by the Dementia Bank and classified them using machine learning to achieve an F-measure of 0.74 (20).

Fraser et al. performed an image description task on mildly cognitively impaired (*n* = 37) and normal (*n* = 37) participants. Linguistic features were extracted from the obtained speech data and discriminated, resulting in an area under the curve (AUC) value of up to 0.87 (21).

Recently, methods using deep learning have also been proposed, and Klekar et al. used the Dementia Bank to classify people with dementia and healthy people and reported that they achieved 91% accuracy (22). In a review article, we surveyed studies that experimented with image description tasks on corpora (23). As GPU performance has improved, it has become easier to construct computationally expensive models, and high accuracy can be expected for sentence-level parsing.

### 2.2. Tasks contributing to classification accuracy and the feasibility of their application

Shihata et al. (24) created a corpus of 60 (30 men and 30 women) older adults (GSK2018-A) with a control group, linking their speech data to a stimulus task and the results of a cognitive function test on the MMSE. Three types of stimulus tasks were included: an episodic task in which participants talked about personal events, an explanatory task for a Cookie Theft Picture, and a task in which participants watched and described a NAIST DOGS animation (produced by Nara Institute of Science and Technology). The episodic task included “1a. recent sad event,” “1b. when it occurred,” “2. recent events that made you feel anxious,” “3. recent events that made you angry,” “4. recent event that made you feel disgusted,” “5. A recent surprising event,” “6a. a recent pleasant event,” “6b. when did it happen?,” “7. what are you passionate about?” and “8. Who do you admire and respect?,” and one image and animation for a total of 12 tasks. Participants were instructed to speak freely for 1–2 min in response to each task question, and their utterances were recorded as audio accompanied by manually transcribed text data.

In a previous study by Igarashi et al. (25) using a corpus created by Shihata et al., the binary classification of a healthy older group and an MCI older group by natural language processing showed high accuracy in the picture description task, the animation description task, and some episodic tasks.

However, the picture description and animation description tasks require a display during the conversation, thus, it is difficult to consider these tasks as natural conversations. On the other hand, in psychiatry and geriatrics, intake interviews are conducted with patients to obtain relevant information in order to provide comprehensive support in treatment. Intake interviews are the most common type of interview in clinical psychology, occurring when a client first comes to a clinician for help (26). The interviews are often conversational in nature and considered beneficial to both parties, as the inclusion of personal conversation topics can lead to a mutual understanding of the interviewer’s and patient’s communication styles (27).

In practice, interviews are conducted by nurses, psychologists, and social workers about the patient’s life history and current living situation, and this information is shared with the physicians and medical teams for smooth treatment and discharge planning. In some cases, the examiner’s findings on the patient’s cognitive function are also included, but these are findings based on experience and are often difficult to extract for nurses and psychologists who have just been assigned to the patient’s care.

If the cognitive function could be estimated mechanically from conversations conducted with patients in practice, it would reduce the burden on hospital staff and patients. However, when considering their constant and widespread use in hospitals, it is necessary to develop question items that can be used universally for any patient. Therefore, this study aims to develop interview items that can be used in hospital practice.

In addition, we will verify the degree of classification accuracy that can be expected from the data collected through interviews. Although previous studies have shown that it is possible to distinguish between an older group and an MCI older group based on MMSE results with an accuracy of 89% or higher, it is not yet known whether the same level of accuracy can be achieved when cognitive decline has progressed beyond MCI. Therefore, we will also develop and validate the accuracy of a natural language processing model capable of multilevel classification of three types of dementia: moderate dementia with an MMSE score of 20 or less, mild dementia with an MMSE score of 21–23, and MCI with an MMSE score of 24 or more and 27 or less.

## 3. Materials and methods

### 3.1. Creation of life history interview items for the intake interview

#### 3.1.1. Experimental environment

The experiment was conducted in a laboratory at the University of Tokyo Hospital. The participant and interviewer sat face-to-face at a desk in the examination room, and questions were asked. Audio and time-lapse images were recorded using a recorder and a small camera [Gopro hero10 (28)].

**Figure 1:**
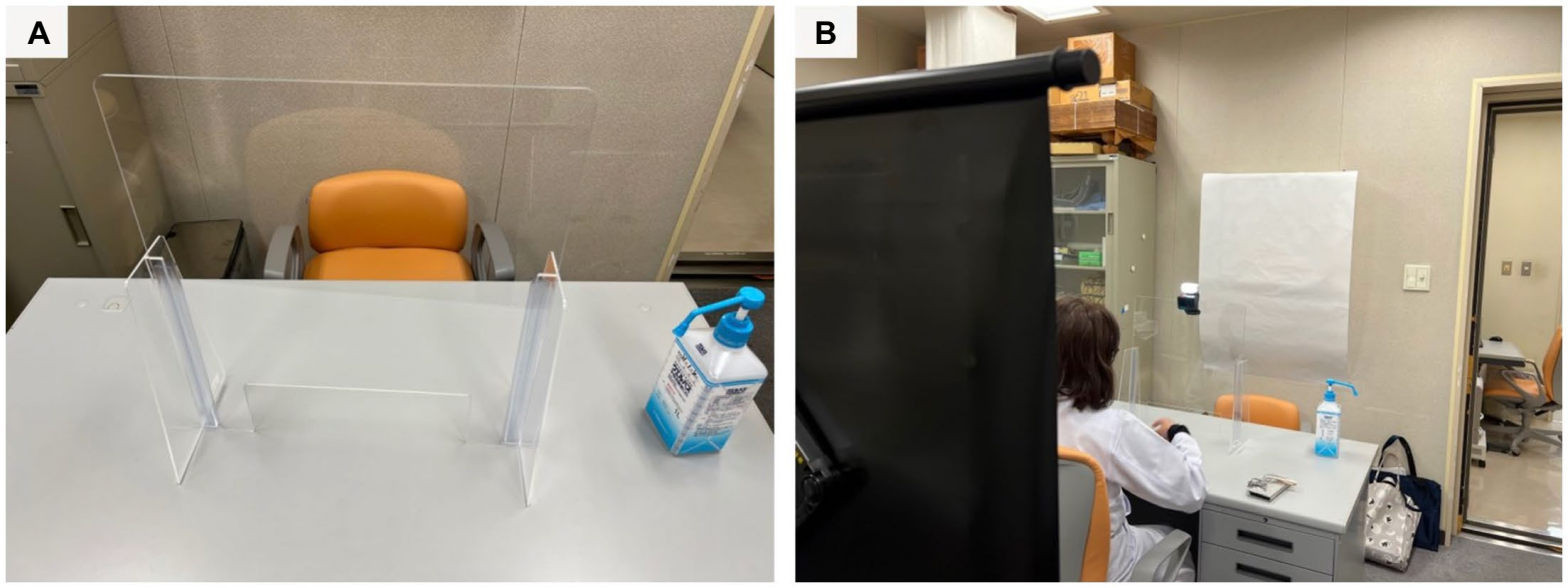
**(A)** A table is partitioned by an acrylic board. **(B)** The questioner sits on the far side and the participant sits on the front side.

To prevent COVID-19 infection, as the interviews were conducted in the period when the pandemic was abating, the interviewer and the questioner wore a face guard and the participants’ hands were disinfected when they entered the room. The room was ventilated with a circulator, and an acrylic board was used as a partition between the participant and interviewer. In addition, the desks and chairs used were disinfected with alcohol spray and paper napkins after the participants left the room. Figure 1 shows a psychological testing room in a hospital.

#### 3.1.2. Method of creating life history interview items for intake interviews

The life history interview items required content that could be used in actual hospital practice. Therefore, interview items were developed according to the following protocol:

- The author attended an intake interview with a psychologist at the University of Tokyo Hospital and surveyed the questionnaire items.
- Items deemed unimportant or duplicated from the questionnaire items were deleted to develop a preliminary draft.
- The draft was checked by five licensed psychologists working at the University of Tokyo Hospital, who made additions and revisions, and changed the order of the questions as required.
- After confirmation by the authors and supervisors, a final version was prepared.

The questionnaire consisted of 30 questions in five categories. The categories were: (1) process before coming to the hospital, (2) life history, (3) ordinary life, (4) interests and concerns, and (5) plans for the rest of the day, with questions related to each category included in the lower tiers. Table 1 shows the questions included in each category.

**Table 1:** Intake interview questions created.

(1) *Process before coming to the hospital*
Q1. Where is your home?
Q2. How long did it take you to get here today?
Q3. After you left your home, how did you come here?
Q4. What time did you leave home to come to the hospital today?
(2) *Life history*
Q5. Where were you born?
Q6. Do you have any siblings (if so, how many?)
Q7. Which elementary school did you attend?
Q8. What did you do after elementary school? (Which junior high school did you attend?)
Q9. What did you do after graduating junior high school? (Which high school did you attend?).
Q10. What do you do for work? (Do you have any memorable stories?)
Q11. Are you married? (When was your wedding?)
Q12. Do you have any children? (Where do your children live?)
(3) *Normal life*
Q13. How do you usually spend your time? (Please tell us your approximate weekly schedule.)
Q14. What time do you get up in the morning and go to bed?
Q15. How often do you go out? (Where do you go most often?)
Q16. Do you bathe every day? (Do you bathe in a bathtub?)
Q17. How do you prepare your meals? (Do you eat three meals a day?) / What did you eat last night?
Q18. How do you clean your house? (How often do you clean your house?)
Q19. How do you do your laundry? (How often do you do it?)
(4) *Interests*
Q20: What news have you been interested in on TV or the Internet recently?
Q21: Please tell me about a sad event that happened to you recently.
Q22: Please tell me about a recent unsettling event.
Q23: Tell me about a recent event that made you angry.
Q24: Tell me about a recent event that made you feel bad.
Q25: Tell me about a recent event that surprised you.
Q26: Tell me about a recent happy event that happened to you. When did it happen?
Q27: Tell me about someone you admire.
Q28: What are you passionate about these days?
(5) *Plans for the rest of the day*
Q29: What are your plans for the rest of the day? (How will you get home?)
Q30: When was the date of your last visit?

#### 3.1.3. Interviewer attitude, reactions, and additional questions

The interviewer was a licensed psychologist; however, he is not a hospital staff member. Therefore, there was no prior relationship between the questioner and the study participants, as they were completely new to each other. As for the interviewer’s attitude, the interviewer was masked, but nonverbal expressions such as nodding and eyes smiling were used to establish a comfortable situation for speaking.

To ensure that the conversation did not end with a short response after asking a question, the interviewer implemented two types of reactions: one was to repeat the information given by the participant as mirroring, to encourage the participant to continue talking after their initial response. The second was a reaffirmation of the participant’s response of “nothing in particular,” followed by the question, “If you had to give a strong answer, what would it be?”

### 3.2. Evaluation

#### 3.2.1. Participants

Participants were recruited from August to September 2022, on the inclusion conditions that they were older people aged 65 years or above, had been diagnosed with dementia by a physician and were able to provide their consent after explaining the outline of the study. As a result, 32 people (9 men and 23 women) between the ages of 72 and 91 years participated in the experiment. A consent form was obtained from the patient if they came to the hospital alone or from a relative accompanying the patient. This study was approved by the Ethical Review Committee of the University of Tokyo Hospital.

#### 3.2.2. Assessment tests

The test content was divided into (a) MMSE, (b) GDS, and (c) life history interview as part of the intake interview. The MMSE is one of the most common assessment methods for detecting dementia. It is a 30-point cognitive function test consisting of 11 items: time and place perception, immediate and delayed wordplay, calculation, object calling and sentence recitation, 3-step verbal command, written command, and graphic imitation. In the MMSE, 23 points or less indicates potential dementia (sensitivity of 81%; specificity of 89%), and 27 points or less indicates potential MCI (sensitivity of 45–60%; specificity of 65–90%) (29–32).

Since sequelae of cerebrovascular disease, lacunar infarction, moderate white matter lesions, parkinsonism, and hypothyroidism can affect speech fluency, we always conduct a set of medical examinations by dementia specialists as well as psychological testing. The dementia specialist has confirmed that the participants selected for this study have cognitive decline and the selected participants did not show any language impairment in other factors. However, it has been reported that the results of cognitive function tests show no significant changes within 3 months. Therefore, if the patient had undergone a cognitive function test at the same hospital within 3 months, the test was omitted, and the most recent test result was referred to in order to reduce the patient’s burden.

The GDS is a screening test used to assess depression in older adults. It was administered to ascertain which participants were above the threshold, as it is known that the amount of conversation is reduced when a person is depressed.

The MMSE results showed that 12 participants had moderate dementia with scores of 20 or less, eight participants had mild dementia with MMSE scores between 21 and 23, and nine participants had mild cognitive impairment (MCI) with MMSE scores between 24 and 27. The GDS results also showed that 27 patients scored below the GDS cutoff of 7 points, while two patients scored higher than the cutoff. However, given that the diagnosis of depression was not made by a specialist’s examination immediately after the test, these two patients were not excluded from the study.

#### 3.2.3. Classification methods

Generalized language models pre-trained on large corpora achieve excellent performance in natural language tasks. Although many pre-trained transformers for English have been published, there are not many model options available, especially for Japanese texts. In this study, we used Bidirectional Encoder Representations from Transformers (BERT), a pre-trained Japanese language model that is considered a ubiquitous baseline for NLP experiments. BERT is a type of neural network based on an architecture called Transformer (33) and provides powerful encoding for sentences and text using word embedding. The representation of a word as a vector of fixed length is called word embedding and it is now possible to obtain multiple distributed representations from a single word by using BERT. Speech data was transcribed manually due to the possibility of transcription errors when using ASR; before loading into BERT, the data were shuffled and split into training data, validation data, and test data.

The model used in this study is a pretrained Japanese BERT model published by the Inui/Suzuki Laboratory of Tohoku University. The models consist of 12 layers, 768 dimensions of hidden states, and 12 attention heads. As the parameters for fine-tuning in this study, we set the batch size as 1, the learning rate as 2e-5, and the number of epochs as 5 based on previous studies using similar methods. In addition, because there were several sentences in the dataset that exceeded 256 characters, sentences longer than 256 were truncated.

Training data, validation data, and test data are split at a ratio of 8:1:1. The splitting is performed using scikit-learn. The classification methods used for multi-level and binary classification are essentially the same. As the target genre, [moderate][mild][MCI] is given as teacher data, and the training data portion is trained. The language processing model called BERT is trained while masking (replacing with expressions such as ****) the original teacher data, which improves generalization performance for various applications.

#### 3.2.4. Dealing with unbalanced data

Obtaining data from patients with dementia is difficult owing to issues of research ethics surrounding obtaining their consent, and collecting a large number and variety of cases is not always possible. In this study, the data were unbalanced: 12 patients had moderate dementia with MMSE scores of 20 or less, eight patients had mild dementia with MMSE scores between 21 and 23, and nine patients had mild cognitive impairment (MCI) with MMSE scores between 24 and 27. The sample sizes should be comparable because different sample sizes make it difficult to analyze the methods and tasks that may have contributed to the classification results.

Undersampling is the simplest method for dealing with unbalanced data, but it leaves the issue of the total amount of data being small (34). One oversampling method that adjusts minority data to the majority is data balancing through data augmentation (35). This method has been particularly successful in the field of imaging, where similar data are augmented by inverting, scaling, and various other methods. It has the advantage of enabling improved classification accuracy on small datasets. Therefore, we augmented the dataset using a technique called easy data augmentation (EDA), which has been shown to be effective in natural language processing (36).

Easy data augmentation consisted of four algorithms. Synonym replacement randomly selects a word in a sentence and replaces it with one of a list of synonyms for that word (excluding stop words). Random Insertion randomly selects a word in a sentence and randomly inserts it at a different position in the sentence (excluding stop words). Random swap randomly selects two words in a sentence and swaps their positions. Random deletion deletes a word in a sentence with probability p (Table 2).

**Table 2:** List of easy data augmentation algorithm.

*Synonym replacement:*	A word in a sentence is randomly selected and replaced with one of the synonyms for that word. The stop words were excluded from the analysis.
*Random insertion:*	Randomly select a word in a sentence and randomly insert it into another position in the sentence. The stop words were excluded from the analysis.
*Random swap:*	Randomly select two words in a sentence and swap their positions.
*Random deletion:*	Randomly Deletes a word in a sentence with probability p.

For the list of synonyms that needed to be replaced, we used the Japanese WordNet proposed by Isahara et al. (37). The percentage of EDA in each algorithm is represented by the parameter α. The value of α was set to 0.05, which was recommended for this amount of data in the original article because a large value would reduce accuracy (Table 2).

A list of stopwords is necessary for augmentation. Some sentences may contain words that do not make sense when the terms are augmented, and these words can be excluded from text augmentation. Slothlib provided by Oshima et al. cannot be used as it is (38). Slothlib is a Japanese word list proposed by Oshima et al. of Kyoto University. Originally, it was an exclusion item to effectively retrieve information on the web and its associated character code identification, existing clustering algorithms, and web search API services (what words are searched); however, in the field of Japanese natural language processing, it is now often set as a stop word when performing data augmentation. According to previous studies, people with dementia are known to use pronouns more frequently, and pronoun use should not be excluded from augmentation (24). Therefore, as suggested by Igarashi et al. (25), 288 unnecessary words for text augmentation in Japanese, excluding pronouns, were set as stop words.

## 4. Results

From the 12 participants with moderate dementia, 334 responses were obtained. The total number of words was 36,734, with an average of 109.98 words per response. The average duration of silence was 6.15 s.

From the eight participants in the mild dementia group, 234 responses were obtained. The total number of words was 25,647, and the average number of words per response was 109.60. The average duration of silence was 2.02 s.

From the nine participants in the MCI group, 256 responses were obtained. The total number of words was 28,423, with an average of 107.26 words per response. The average duration of silence was 1.93 s.

### 4.1. Multi-level classification results

A total of 660 training data, 82 validation data, and 82 test data are included when multi-level classification is used. The results of the validation with the model of multi-level classification which assigns the three groups showed that the correct answer rate in the training data was 0.919, the correct answer rate in the validation data was 0.530, and the correct answer rate in the test data was 0.405.

### 4.2. Binary classification results

For moderate and mild binary classification, 456 training data, 56 validation data, and 56 test data are included. The results of the validation with the binary classification model, which allocates the group with moderate dementia and the group with mild dementia, showed that the correct answer rate in the training data was 0.821, the correct answer rate in the validation data was 0.482, and the correct answer rate in the test data was 0.488.

For the binary classification of moderate and MCI, 472 training data, 59 validation data, and 59 test data are included. The results of the validation with the binary classification model to sort moderate dementia and MCI groups were as follows: the correct answer rate in the training data was 0.956, the correct answer rate in the validation data was 0.633, and the correct answer rate in the test data was 0.767.

For the binary classification of mild and MCI, 392 training data, 49 validation data, and 49 test data are included. The results of the validation with the model of binary classification to separate the mild dementia group and the MCI group were as follows: the correct answer rate in the training data was 0.965, the correct answer rate in the validation data was 0.660, and the correct answer rate in the test data was 0.700.

### 4.3. Multi-valued classification results (with augmentation)

**Figure 2:**
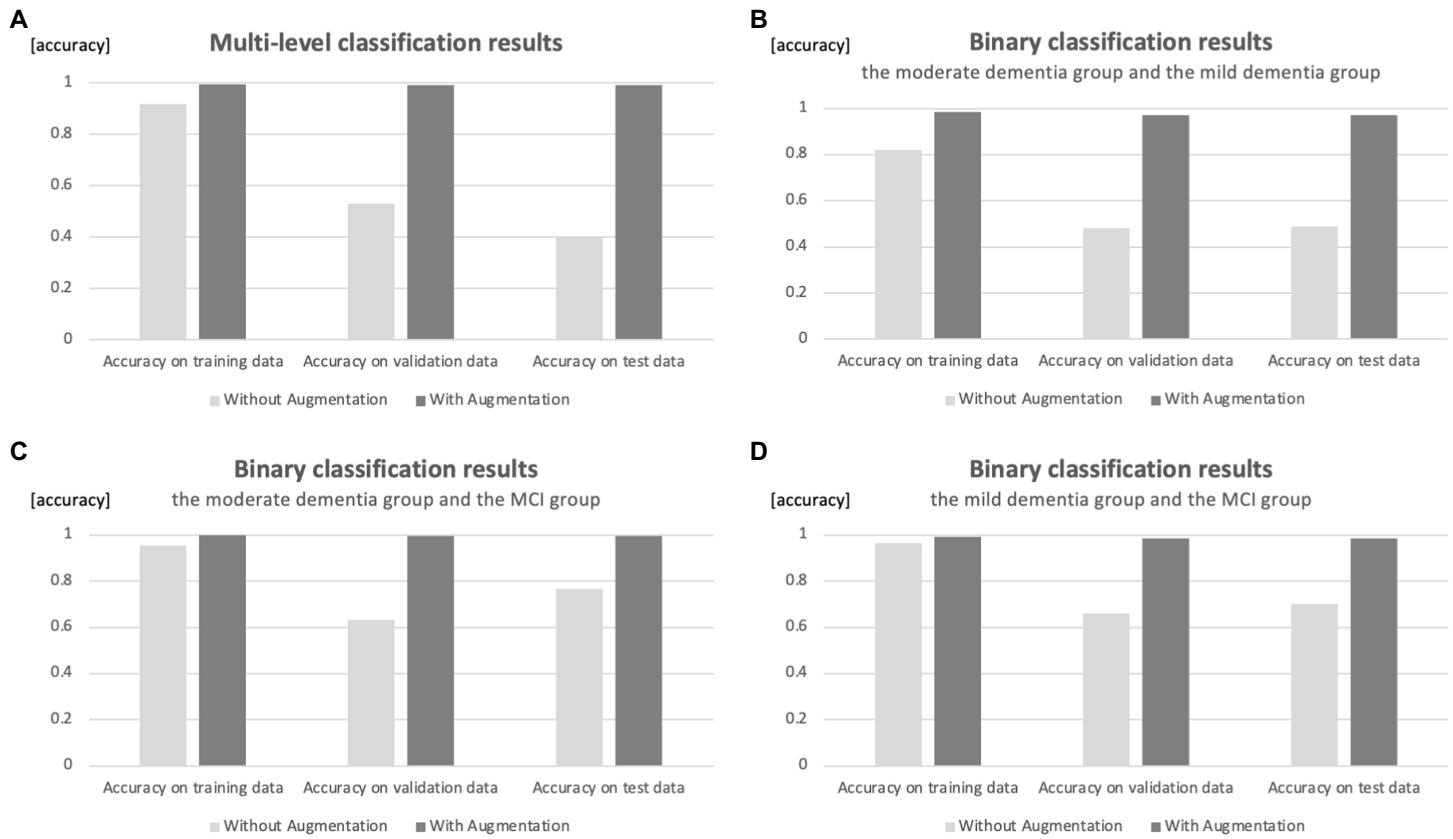
Comparison of accuracy results with and without augmentation. **(A)** Classification of three groups. **(B)** Binary classification of moderate and mild dementia groups. **(C)** Binary classification of moderate dementia and MCI groups. **(D)** Binary classification of mild dementia and MCI groups.

In the case of augmentation, the number of data for each group is increased until it reaches 5,000. This means that when multi-level classification is used, 12,000 training data, 1,500 validation data, and 1,500 test data are included. The results of the validation with the model of multi-level classification, which assigns three groups, showed that the correct answer rate was 0.994 for the training data, 0.992 for the validation data, and 0.991 for the test data. Figure 2 shows a comparison of accuracy results with and without augmentation.

### 4.4. Binary classification results (with augmentation)

For binary classification, 8,000 training data, 1,000 validation data, and 1,000 test data are included for each group’s classification. The results of the validation with the binary classification model that assigns the group with moderate dementia and the group with mild dementia showed that the correct answer rate in the training data was 0.984, the correct answer rate in the validation data was 0.971, and the correct answer rate in the test data was 0.972.

The results of the validation with the binary classification model to sort the moderate dementia and MCI groups were as follows: the correct answer rate in the training data was 0.999, the correct answer rate in the validation data was 0.997, and the correct answer rate in the test data was 0.996.

The results of the validation with the binary classification model, which allocates mild dementia and MCI groups, showed that the correct answer rate in the training data was 0.991, the correct answer rate in the validation data was 0.987, and the correct answer rate in the test data was 0.985.

### 4.5. Differences in the text data of each group

The number of words per response in the three groups was 109.98, 109.60, and 107.26 for moderate dementia, mild dementia, and MCI groups, respectively. The number of words per response to the same question in the three groups indicated a tendency to become more verbose as cognitive function declined, but the differences between the groups were small. The results of a t-test with no correspondence among the three groups showed no significant differences.

On the other hand, the average number of seconds of silence per response in the three groups was 6.15 s for the moderate dementia group, 2.02 s for the mild dementia group, and 1.93 s for the MCI group. The average number of silent seconds per response to the same question for the three groups tended to increase as cognitive function declined. In addition, the results of the *t*-test without correspondence among the three groups showed significant differences.

This suggests that at least for the results of the intake interview-based open queries used in this study, the previous study’s finding that conversations become more verbose as cognitive function declines is related to an increase in the number of silent seconds in the conversation rather than the number of words in the response.

## 5. Discussion

### 5.1. Relationship between classification accuracy and augmentation

The accuracy of the test data in the multi-value classification results was 0.405 without augmentation and 0.991 with augmentation.

The accuracy of the test data in the results of the binary classification without augmentation was 0.488 for moderate dementia and mild dementia groups, 0.767 for moderate dementia and MCI groups, and 0.700 for mild dementia and MCI groups. This suggests that without augmentation, it was possible to classify moderate dementia (or mild dementia) and MCI groups with an accuracy of over 70%; however, it was difficult to classify moderate dementia and mild dementia groups.

On the other hand, the accuracy of the test data in the binary classification with augmentation was 0.972 for moderate dementia and mild dementia groups, 0.996 for moderate dementia and MCI groups, and 0.985 for mild dementia and MCI groups.

In the case of no augmentation, the correct response rate for both the multi-level and binary classification cases was high for the training data but low for the validation and test data. This is thought to be due to overlearning, which results in excessive adaptation to the training data.

In both multi-level and binary classification cases, the accuracy of the case with augmentation was significantly higher than that without. The accuracy of both multilevel and binary classification cases with augmentation exceeded 97%, suggesting that augmentation may be useful as reference information in cases where the MMSE or other tests cannot be performed.

### 5.2. Future study

There is a possibility of overfitting due to the small data size. For the reliability of the language processing model, it would be desirable to revalidate the model with an increased sample size. We plan to recruit community-dwelling older adults to test a similar questionnaire and MMSE in future. We believe that a study of the four-value classification of MCI, mild dementia, and moderate dementia, including healthy older adults, could provide additional information on reliability. However, we are using a model that has been fully trained in Japanese by BERT in a fine-tuned form. In the study by Marius et al., the accuracy remained almost unchanged in the range of training loss from 10^−5^ to 10^−1^, indicating that overfitting (overfitting to the training data) did not occur during fine-tuning. Nevertheless, more data is important to reduce the effect of overtraining.

As mentioned in related studies, using existing publicly available datasets in Japanese, the classification performance of healthy older adults and MCI was higher when using the picture description task, the animation description task, and some episodic tasks. There were 30 questions in total in the questionnaire we developed, all of which were used for this classification. However, there are five categories of questions, and it is believed that some of the categories and individual questions significantly enhance the accuracy of the classification, while others, on the contrary, reduce it.

Although the average interview with participants in our study lasted about 1 h, it is necessary to conduct interviews for a shorter duration of time to reduce the burden on hospital staff and patients, especially in hospital settings. Therefore, a detailed analysis of each question item in order to create a more refined questionnaire will be a future task.

Regarding the classification of silence time, we believe that image data drawn with MFCC or Mel spectrogram of the original acoustic features will leave better results than features extracted with text. Since those analyses are different from the accuracy verification by augmentation in this study, we hope to be able to verify them in future studies.

### 5.3. Use case

As described in related studies, intake interviews are conducted in many psychiatry departments. As a use case, when conducting an intake interview with a new patient, a pin microphone or similar device is provided to record data. After the intake interview, the voice data are converted to a text file on a PC, and this model can be used to check the classification results in a few 10 of seconds. If the intake interview is conducted prior to the physician’s visit, the physician can review the results at that time for additional consultation.

On the other hand, in terms of rigor, the proposed system, like MMSE, is not a screening system. MRI is needed to pinpoint phenomena in the brain more precisely. Therefore, rather than considering this system as a comparative system that aims to completely replace MRI, it may be better to consider it as a system that can be applied to outreach activities in remote medical areas and home visits where expensive medical equipment resources are scarce.

## 6. Limitation

In this study, the transcription of statements was performed manually to prevent errors in the transcription process. However, hand transcription is unrealistic for clinical use. Therefore, if this is to be fully automated, automatic speech recognition (ASR) should be utilized; however, it is not known whether the same accuracy can be achieved if transcription errors that occur in such cases are included. In addition, the use of ASR may be affected by noise and in-building broadcasts. However, these problems may be resolved using a pin microphone that picks up the volume only around the patient’s mouth.

The small size of the data and its split method for training and validation is also a limitation of this study. With the contribution of this study, future research collaboration is encouraged to expand the sample size. As explained in the Methods section, there were 29 participants in this study, 12 people had moderate dementia, 8 people had mild dementia, and 9 people had MCI. Therefore, it is clear that our data cannot be split 8:1:1 when divided by the number of participants.

Since data within the same group are considered to have the same sentence feature, it is natural that the response data from the same participant could be in either the training or validation dataset. However, there is no doubt that it would be best if the sample size could be increased. Validation of only one data from each group with the large dataset should be clarified in future studies.

## 7. Conclusion

In this study, we developed an interview framework and natural language processing model for estimating cognitive function, based on an intake interview with psychologists in a hospital setting. The interview items were prepared by the author, who witnessed the psychologist’s intake interview and deleted unimportant or duplicated items from the questionnaire.

The questionnaire consisted of 30 questions in five categories. The categories were as follows: (1) process before coming to the hospital, (2) life history, (3) ordinary life, (4) interests and concerns, and (5) plans for the rest of the day, with questions related to each category included in the tier below it.

The Japanese version of BERT, pre-trained on a large corpus, was used as the natural language processing model for estimating cognitive functions. However, because of the small number of study participants, it was difficult to achieve accuracy simply by using the raw data without modification for training; therefore, EDA was conducted to increase the text data using four different methods. During EDA, augmentation was performed by excluding terms that were thought not to be characterized by a cognitive decline as stop words.

To evaluate the feasibility of the developed interview items and the accuracy of the natural language processing model, we recruited participants with the approval of the University of Tokyo Hospital and obtained the cooperation of 29 participants (7 men and 22 women) aged 72–91 years. Three types of tests, MMSE, GDS, and a life history interview, were conducted at the laboratory of the University of Tokyo Hospital. The examinations were recorded using a recorder and a small camera (Gopro hero10) for audio and time-lapse images.

The results of the MMSE showed that 12 patients had moderate dementia with an MMSE score of 20 or less, eight patients had mild dementia with an MMSE score between 21 and 23, and nine patients had mild cognitive impairment (MCI) with an MMSE score of 24 or more and 27 or less. Therefore, based on the MMSE results, a multilevel classification model was created to classify the three groups, and a binary classification model was used to sort the two groups. For each of these models, we tested whether the accuracy would improve when text augmentation was performed.

The accuracy in the multi-level classification results for the test data was 0.405 without augmentation and 0.991 with augmentation. The accuracy of the test data in the results of the binary classification without augmentation was 0.488 for moderate dementia and mild dementia groups, 0.767 for moderate dementia and MCI groups, and 0.700 for mild dementia and MCI groups. This suggests that without augmentation, it was possible to classify moderate dementia (or mild dementia) and MCI groups with an accuracy of over 70%; however, it was difficult to classify moderate dementia and mild dementia groups.

In contrast, the accuracy of the test data in the augmented binary classification results was 0.972 for moderate dementia and mild dementia groups, 0.996 for moderate dementia and MCI groups, and 0.985 for mild dementia and the MCI groups.

Comparing the accuracy with and without augmentation for both multilevel and binary classification, the accuracy increased significantly with augmentation. The accuracy of both multilevel and binary classification cases with augmentation exceeded 97%, suggesting that augmentation may be useful as reference information in cases where the MMSE or other tests cannot be administered.

## Data availability statement

The original contributions presented in the study are included in the article/supplementary material, further inquiries can be directed to the corresponding author.

## Ethics statement

The studies involving human participants were reviewed and approved by the Ethics Review Committee, The University of Tokyo Hospital. The patients/participants provided their written informed consent to participate in this study.

## Author contributions

TI, YU-K, TK, MA, and MN contributed to the conception and design of the study. TI organized the database, performed the statistical analysis, and wrote the first draft of the manuscript. All authors contributed to the manuscript revision, read, and approved the submitted version.

## Funding

The authors declare that this study received funding from the Japan Agency for Medical Research and Development (AMED) (grant number: 22he2002025j0002). The funder was not involved in the study design, collection, analysis, interpretation of data, the writing of this article or the decision to submit it for publication.

## Conflict of interest

The authors declare that the research was conducted in the absence of any commercial or financial relationships that could be construed as a potential conflict of interest.

## Publisher’s note

All claims expressed in this article are solely those of the authors and do not necessarily represent those of their affiliated organizations, or those of the publisher, the editors and the reviewers. Any product that may be evaluated in this article, or claim that may be made by its manufacturer, is not guaranteed or endorsed by the publisher.
